# Whole-cell display of *Pyrococcus horikoshii* glutamate decarboxylase in *Escherichia coli* for high-titer extracellular gamma-aminobutyric acid production

**DOI:** 10.1093/jimb/kuab039

**Published:** 2021-06-28

**Authors:** Sivachandiran Somasundaram, Jaehoon Jeong, Ashokkumar Kumaravel, Soon Ho Hong

**Affiliations:** Department of Chemical Engineering, University of Ulsan, 93 Daehak-ro, Nam-gu, Ulsan 44610, Republic of Korea; Department of Chemical Engineering, University of Ulsan, 93 Daehak-ro, Nam-gu, Ulsan 44610, Republic of Korea; Department of Chemical Engineering, University of Ulsan, 93 Daehak-ro, Nam-gu, Ulsan 44610, Republic of Korea; Department of Chemical Engineering, University of Ulsan, 93 Daehak-ro, Nam-gu, Ulsan 44610, Republic of Korea

**Keywords:** Cell-surface display, Glutamate decarboxylase, Gamma-aminobutyric acid, Outer membrane protein, Thermophilic enzyme

## Abstract

We investigated the effect of cell-surface display of glutamate decarboxylase (GadB) on gamma-aminobutyric acid (GABA) production in recombinant *Escherichia coli*. We integrated GadB from the hyperthermophilic, anaerobic archaeon *Pyrococcus horikoshii* to the C-terminus of the *E. coli* outer membrane protein C (OmpC). After 12 hr of culturing GadB-displaying cells, the GABA concentration in the extracellular medium increased to 3.2 g/l, which is eight times that obtained with cells expressing GadB in the cytosol. To further enhance GABA production, we increased the temperatures of the culture. At 60°C, the obtained GABA concentration was 4.62 g/l after 12 hr of culture, and 5.35 g/l after 24 hr, which corresponds to a yield of 87.7%.

## Introduction

The worldwide use of nonbiodegradable polyethylene-based plastics for the past decades has caused serious environmental problems such as plastic island and microplastics. Studies on biodegradable polymers aim to find an alternative. Polyamide 4 or nylon 4, one of the promising biodegradable polymers, is produced by ring-opening polymerization from petroleum-derived 2-pyrrolidone (Kawasaki et al., 2005; Saskiawan 2008). However, 2-pyrrolidone can also be obtained from gamma-aminobutyric acid (GABA), which is produced by the fermentation of genetically engineered microorganisms (Park et al., 2013). GABA is a nonprotein amino acid that acts as a neurotransmitter in the central nervous system in the mammalian brain and has hypotensive, diuretic, and relaxing effects (Boonstra et al., 2015).

GABA is a natural metabolic intermediate in many organisms and can be produced from l-glutamate by glutamate decarboxylase (Park & Oh, 2004). To enhance GABA production in recombinant bacteria, the introduction and overexpression of various glutamate decarboxylases have been investigated in various bacterial strains. Glutamate decarboxylase from *Lactobacillus* is one of the most tested glutamate decarboxylases. Overexpression of *Lactobacillus plantarum* ATCC 14917 glutamate decarboxylase in *Lactobacillus sakei* B2-16 increased the final GABA concentration by 1.4-fold compared with the unmodified strain (Kook et al., 2010). In another study, introducing *L*actobacillus *brevis* glutamate decarboxylase into *Escherichia coli* BL21 (DE3) enhanced GABA production (Lim et al., 2018). Overexpressing rice glutamate decarboxylase in *Bifidobacterium longum* yielded a final GABA concentration of 0.1 g/l from 30 g/l of monosodium glutamate (MSG) (Park et al., 2005). Recently, overexpressing glutamate decarboxylase genes from *Sulfobacillus thermosulfidooxidans* coupled with a two-stage production strategy allowed to produce 9 g/l of methanol-based GABA from *Bacillus methanolicus* (Irla et al., 2017). A synthetic scaffold strategy between GadB and GadC colocalization yielded 5.96 g/l and 5.94 g/l of GABA from 10 g/l of MSG in a GABA aminotransferase knock-out *E. coli* strain (Somasundaram et al., 2017a; Somasundaram et al., 2017b).

The cell-surface display system allows the expression of target proteins, enzymes, or peptides on the bacterial surface by using a membrane-bound protein as an anchor. Surface display systems contain a carrier protein that acts as an anchoring motif and a passenger protein that acts as a target protein. In bacteria, many surface proteins have been investigated and used as an anchoring motif, such as outer membrane proteins, lipoproteins, and S-layer proteins (Georgiou et al., 1997; Jung et al., 1998; Lee et al., 2006). Several outer membrane proteins including OmpA, OmpC, OmpF, OmpS, OprF, PhoE, and LamB were used as an anchoring motif to display various peptides/proteins, and their performance was studied in many biotechnology applications (Xu & Lee, 1999; Benhar, 2001; Samuelson et al., 2002).

Bacterial cell-surface displays have many potential applications such as vaccination, antibody production, biosensor development, bioadsorption, bioconversion, and peptide library screening (Lee et al., 2003; Löfblom, 2011). Recently, cell-surface-displayed recombinant bacteria served as a biocatalyst to produce biofuel and as biosorbents to treat polluted water (Yanase et al., 2010; Wen et al., 2010; Kuroda & Ueda, 2011).

Recent applications of cell-surface display strategies to biorefinery processes have attracted intensive attention. In particular, the display of cellulases on the cell surface of yeast to produce ethanol from cellulose and starch (Tanaka et al., 2012; Yamada et al., 2010, 2011), or the display of Xylanase (XynA) from *Thermomyces lanuginosus* on *E. coli* to degrade xylan. The recombinant *E. coli*-displayed XynA had the highest xylan degradation activity at pH 6.2 and 65°C (Qu et al., 2015).

**Fig. 1.**
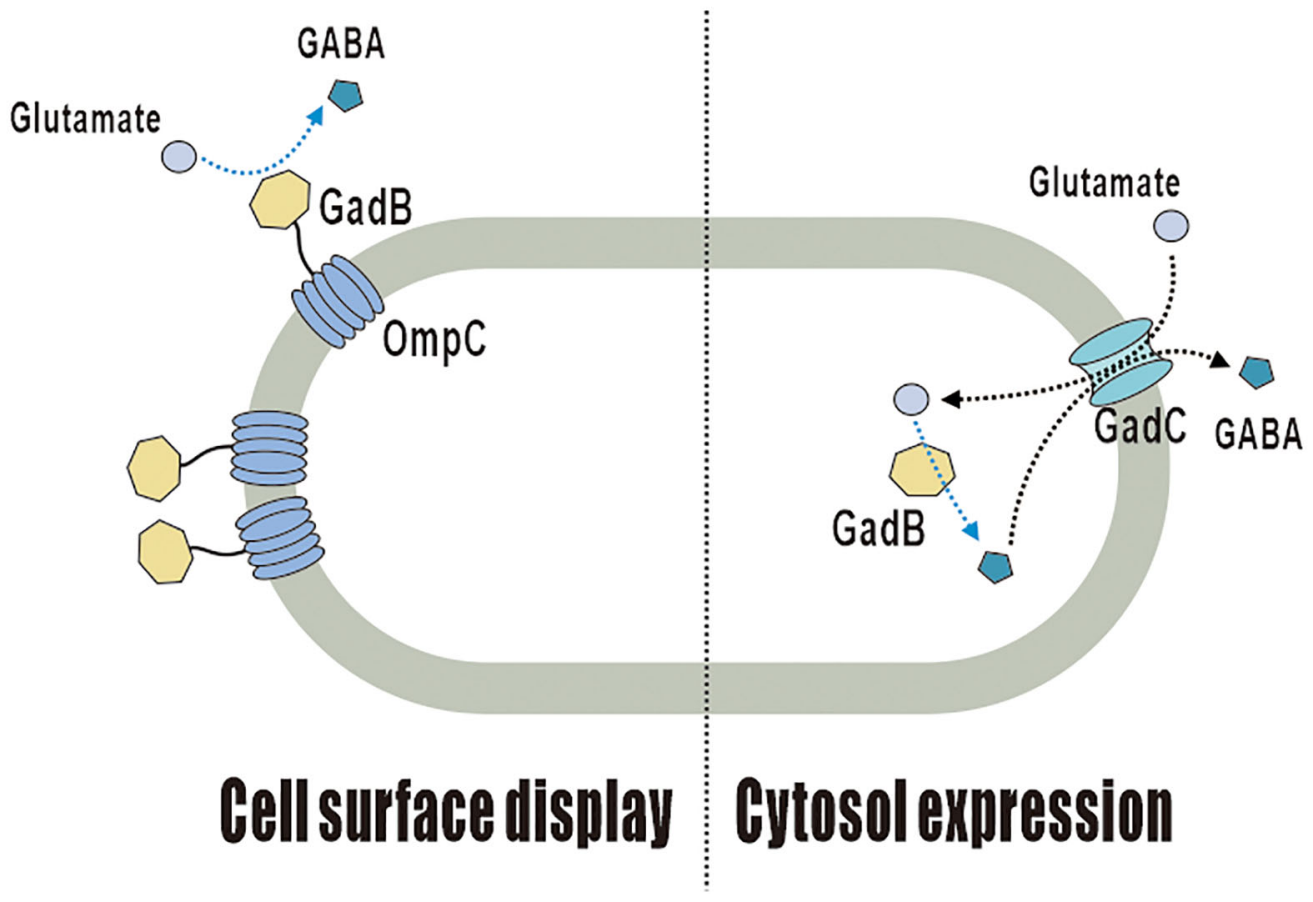
Schematic diagram of GadB surface display and gamma-aminobutyric acid bioconversion in *E. coli.*

In this study, we present a successful cell-surface display system of glutamate decarboxylase in recombinant *E. coli* (Fig. 1). We displayed the glutamate decarboxylase (GadB) from the hyperthermophilic anaerobic archaeon *Pyrococcus horikoshii* on the surface of *E. coli* using OmpC as an anchoring motif for the extracellular production of GABA. To investigate the effect of the display of *P. horikoshii* GadB on GABA production, we tested the *P. horikoshii* GadB-displaying recombinant *E. coli* under various environmental conditions.

## Materials and Methods

### Bacterial Strains, Plasmids, and Medium

We used the *E. coli* XL1-Blue (XB) strain. Table 1 lists the strains and plasmids used in this study. The A pGEMT vector (Promega, Madison, Wisconsin, USA) was used for DNA cloning, and pMAL-p4X (New England Biolabs, Ipswich, Massachusetts, USA) was used to construct the expression plasmids. Chemically competent cells were prepared using a standard procedure and stored at −80°C.

**Table 1.** List of Bacterial Strains and Plasmids Used in This Study

Strain/plasmid	Description	Reference
***Escherichia coli* strains**		
XL1-Blue	SupE44 hsdR17 recA1 endA1 gyrA96 thi relA1 lacF'(proAB^+^lacI^q^lacZΔM15 Tn10 (tet^R^))	Laboratory stock
**Plasmid**		
pGEMT	Ap^R^	Promega (USA)
pMAL-p4X	Ap^R^	NEB^a^
pHB	pMAL containing *gadB* from *E. coli*	Tam et al., 2012
pHBP	pMAL containing *gadB* from *Pyrococcus horikoshii*	Tam et al., 2014
pHPE	pMAL containing *ompC* from *E. coli* and *gadB* from *P. horikoshii*	This work

Recombinant *E. coli* XB was cultured at 37°C and 250 rpm in a Luria-Bertani (LB) broth (10 g/l bacto-tryptone, 5 g/l bacto-yeast extract, and 5 g/l NaCl) supplemented with 100 μg/ml ampicillin sodium salt to select the transformants harboring recombinant plasmids (Sambrook & Russell, 2001).

### Plasmids Construction

The outer membrane protein C (*ompC)* gene from the *E. coli* genome and the glutamate decarboxylase (*gadB*) gene from *P. horikoshii* were amplified from their respective genomic DNA using the Expand high-fidelity polymerase chain reaction (PCR) system (Roche Molecular Biochemicals, Mannheim, Germany). The PCR products were purified using a GENEALL PCR purification kit (General Biosystem, Seoul, Korea) and cloned into the pGEMT vector. Then, the *ompC* gene was cloned into the pMAL-p4X expression vector using the restriction sites NdeI and BamHI, and the *P. horikoshii gadB* gene was cloned downstream of *ompC* using BamHI and HindIII enzyme sites to construct an expression plasmid pHPE. The cloning of genes was confirmed by restriction digestion and DNA sequencing.

### GABA Bioconversion and Analysis

Recombinant *E. coli* strains harboring the pHPE plasmid and pHPB plasmid (Table 1) were cultivated in two phases: (i) whole-cell catalyst preparation and (ii) bioconversion phase. In whole-cell catalyst preparation, recombinant cells were first cultured in 250 ml flasks with 100 ml of LB broth (100 μg/ml ampicillin) at 37°C and 250 rpm. In the bioconversion phase, when the OD_600_ reached 1.2, 10 g/l MSG added in the flask and pH was adjusted to 3.5. GadB gene expression was induced with 0.5 mM IPTG and the cells were incubated at 30°C and 250 rpm for 48 hr. All the experiments were performed in triplicate for GABA production.

The GABA bioconversions were quantitatively analyzed by HPLC using an OptimaPak C18 column (4.6×150 mm) (RS tech Corporation, Daejeon, Korea). The sample preparation was performed as follows: 1 ml samples were taken from the culture every 12 hours to analyze the GABA production. The samples were centrifuged at 12,000 rpm for 5 min. 100 μl of the supernatant was placed in an Eppendorf tube. Next, 200 μl of a 1 M sodium bicarbonate buffer at pH 9.8, 100 μl of 80 g/l dansyl chloride in acetonitrile, and 600 μl of double-distilled water were added to make a 1 ml reaction mixture. The mixture was then incubated at 80°C for 40 min. Then, 100 μl of 20 μl/ml acetic acid was added to stop the reaction. The mixture was then centrifuged at 12,000 rpm for 5 min. Next, the supernatant was filtered through a 0.22 μm Millipore filter and analyzed by HPLC on an Agilent system using UV detection. Separation of the derivatized samples was performed using a binary nonlinear gradient with eluant A (tetrahydrofuran/methanol/50 mM sodium acetate with a pH of 6.2 (5:75:420, by volume)) and eluant B (methanol). The column temperature was set at 30°C and the elution conditions were as follows: equilibration (6 min, 20% B), gradient (20 min, 20–80% B), and cleaning (3 min, 100% B). The flow rate of the mobile phase was 1 ml/min and the absorbance of the samples was detected at a wavelength of 286 nm. The standard curve for GABA was generated from 10 standard solutions (1, 2, 3, 4, 5, 6, 7, 8, 9, and 10 g/l GABA) (Sigma, Missouri, USA) using the same procedure.

## Results and Discussion

### Construction of *P. horikoshii* GadB Display System

Generally, the activity of catalysts (including biological catalysts like enzymes) increases with the temperature. However, the optimum temperature of most of the enzymes is around 37°C, and at higher temperatures, their activity decreases. Therefore, the high-temperature stability of enzymes from extremophiles makes them good candidates as novel catalysts. Thus, in this study, we tested GadB from *P. horikoshii* to enhance the production of GABA.

The novel hyperthermophilic anaerobic archaeon *P. horikoshii* OT3 was isolated in 1998. The optimum growth conditions of this strain are 98°C and pH 7. Its maximum growth temperature is 102°C, and it can survive temperatures as high as 105°C. The complete genome sequence of *P. horikoshii* OT3 was determined in 1998, and it is 1738,505 bp long (Kawarabayasi & Sawada, 1998; González et al., 1998). GadB from *P. horikoshii* is a monomeric protein and has been successfully applied to GABA production (Somasundaram et al., 2017). In this study, we displayed it on the surface of the *E. coli* membranes using OmpC as an anchoring motif to improve GABA production.

OmpC is the major outer membrane protein of *E. coli*. It is highly expressed on the *E. coli* cell surface. The OmpC of *E. coli* is a well-characterized protein with a homotrimer structure and is highly stable against protein denaturation (Baslé et al., 2006). We fused the 383 amino acids long GadB from *P. horikoshii* with the C-terminus region of OmpC.

### GABA Production by Surface Display System

We cultured *E. coli* XB strains harboring a pHPE plasmid to produce GABA. We used the previous reported optimum conditions for *P. horikoshii* GadB, namely, pH 3.5 and 30°C (Somasundaram et al., 2017). To figure out the induction conditions, we added various concentrations of the inducer IPTG (0, 0.1, and 0.5 mM) to the culture medium of the recombinant strain. In the absence of IPTG, the cells produced a small amount of GABA, due to the basal gene expression. At 0.5 mM IPTG, the cells produced a high GABA concentration of 5.1 g/l. This result indicates that surface-displayed GadB successfully produced GABA.

**Fig. 2.**
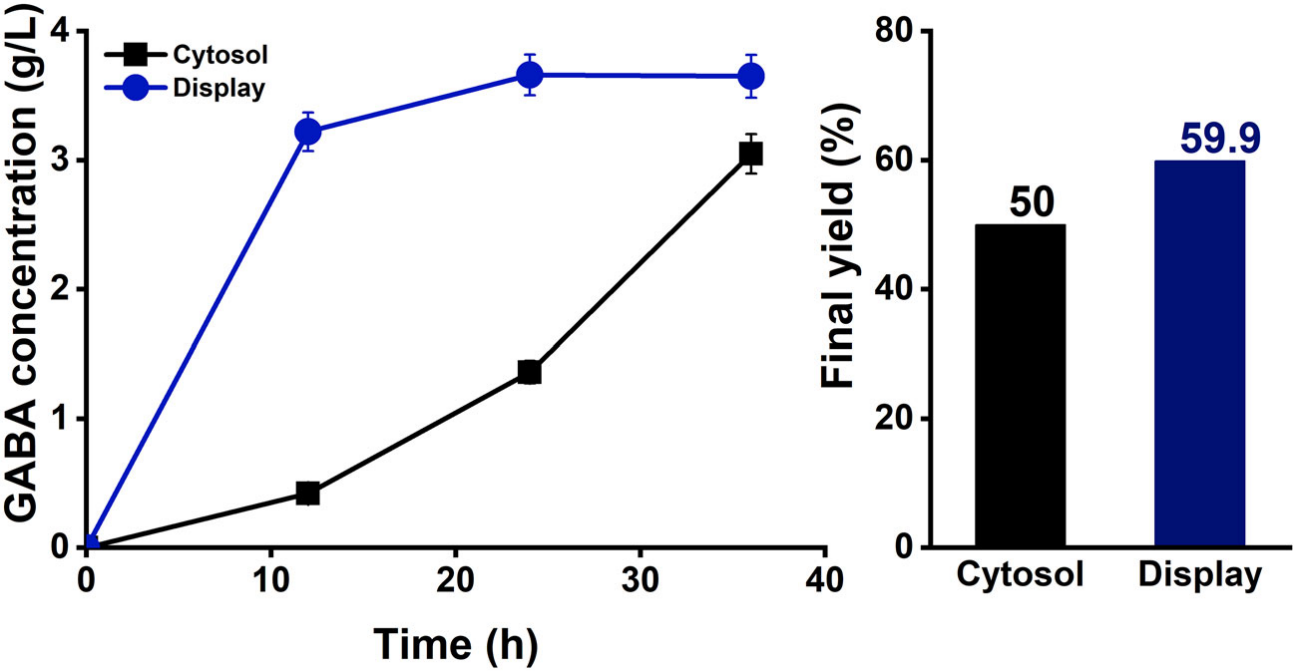
Time profiles of the GABA production rate for 36 hr of bioconversion period between the cytosolic system (■) and surface-displayed system (●), and final yield of GABA percentage was showed between cytosolic and surface-displayed system at 36 hr of bioconversion period.

To evaluate the effect of the cell-surface display of GadB, we compared the GABA concentration-time profiles of displayed GadB and cytosol GadB (Fig. 2). With the recombinant strain harboring the surface-displayed plasmid pHBE, the GABA concentration rapidly increased to 3.2 g/l in 12 hr and gradually raised to 3.65 g/l in 36 hr of bioconversion period (Fig. 2). Meanwhile, the control strain harboring the cytosolic gadB expression plasmid pHBP produced only 0.4 g/l in 12 hr and 3.05 g/l in 36 hr. The notable result observed in the GadB display system, it increased GABA productivity within 12 hr (3.2 g/l in display system compared with 0.4 g/l in cytosol system) of the early biotransformation period.

These results demonstrate the practical advantage of the cell-surface display to improve productivity. The elevated productivity may be due to a shortened GABA reaction step. In the cytosol-expressed GadB strain, glutamate needs to be imported inside the cell to react with GadB and be converted to GABA. Then, GABA is secreted back to the extracellular medium. Whereas with surface-displayed GadB strain, glutamate comes in contact with GadB directly on the cell outer surface and can be converted to GABA in the extracellular medium. Therefore, the conversion of glutamate into GABA by displayed GadB is more efficient, resulting in a higher GABA productivity.

### Enhanced GABA Production by Culture Condition Optimization

**Fig. 3.**
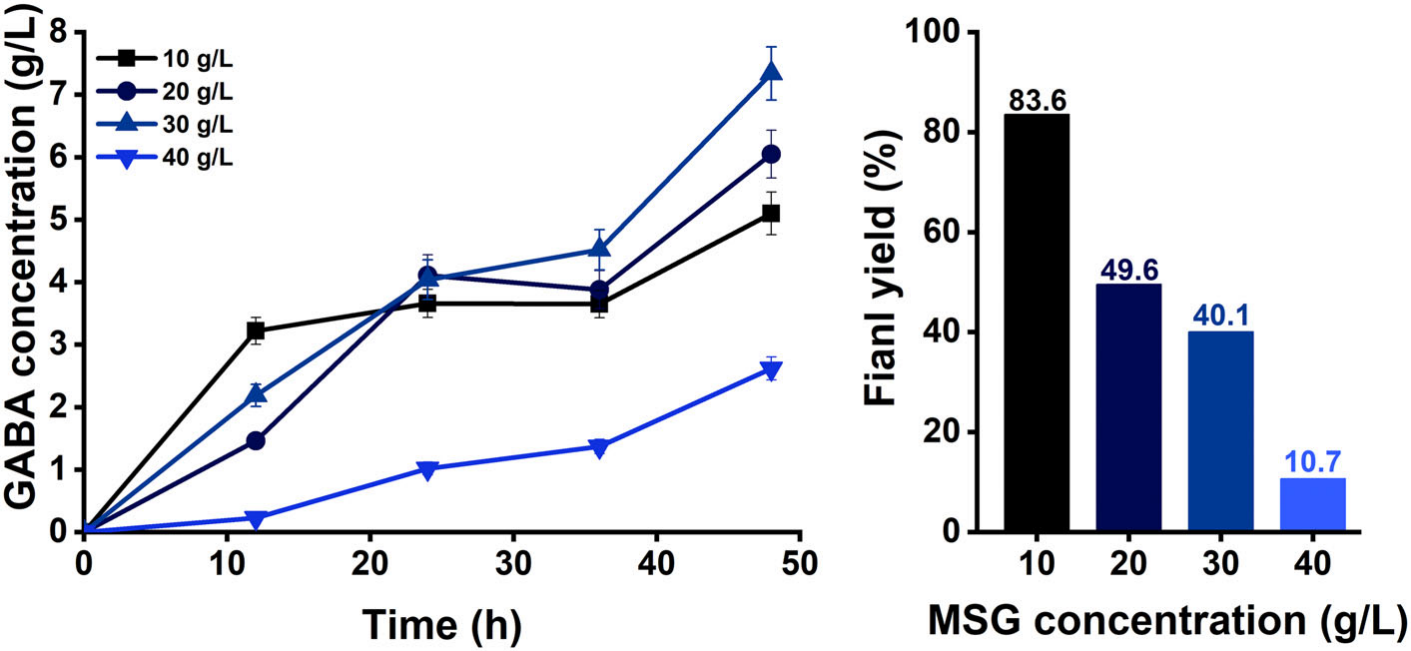
GABA production profiles for 48 hr of bioconversion on various MSG concentrations such as 10 (■), 20 (●), 30 (▲), 40 (▼) g/l by the surface-displayed system and GABA final yield percentage of surface display system at various MSG concentration of 10, 20, 30, and 40 g/l.

Although the surface-displaying recombinant *E. coli* strain successfully produced GABA, the industrialization of the novel GABA process requires a further increase of the GABA titer. To achieve higher GABA concentrations, we investigated the effect of higher concentrations of MSG. Increasing the MSG concentration up to 30 g/l increased the GABA up to 7.34 g/l (Fig. 3). Further increasing of the MSG concentration to 40 g/l reduced the GABA concentration to 2.62 g/l, and at 50 g/l of MSG, the cells failed to produce GABA. While 30 g/l of MSG gave the highest GABA concentration, but the GABA yield was only 40.1%.

### High-Temperature Production of GABA

**Fig. 4.**
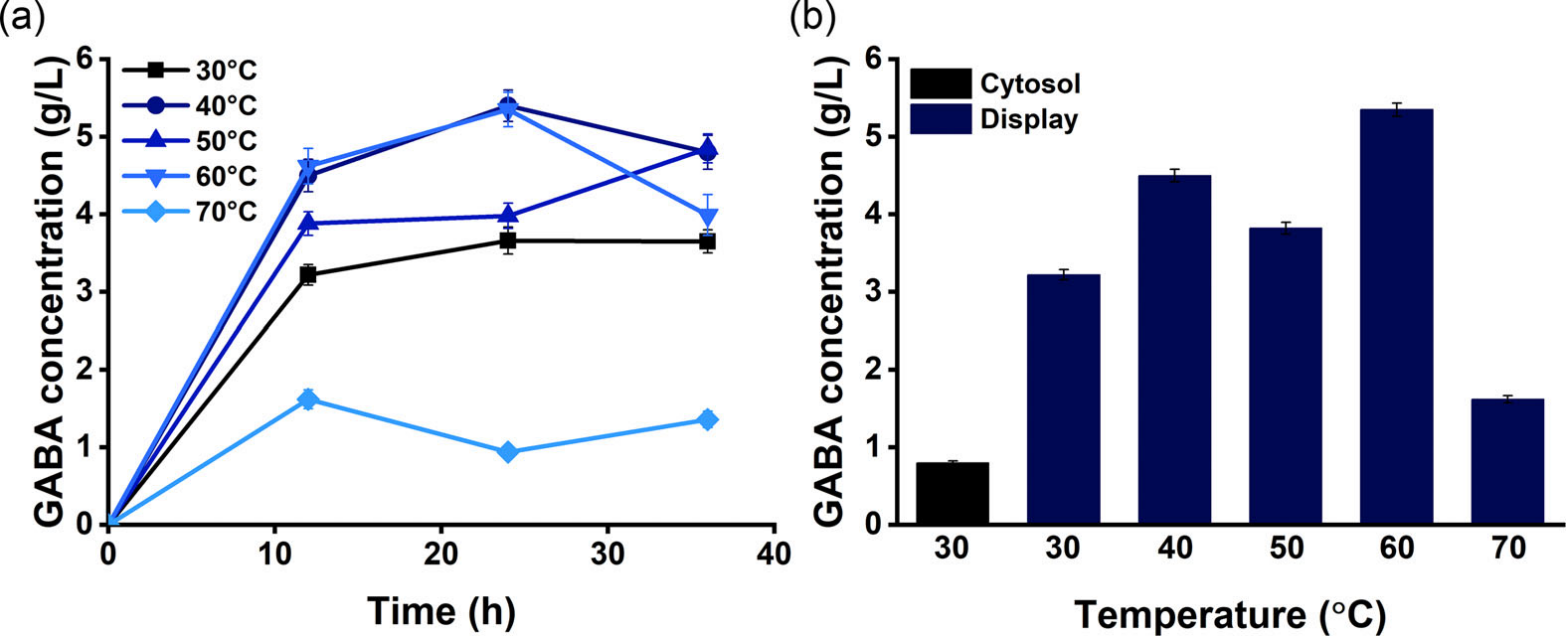
Investigation of surface-displayed thermophilic GadB for GABA production at high temperatures. (a) Time profile of GABA productivity rate for 48 hr at various temperature ranges such as 30 (■), 40 (●), 50 (▲), 60 (▼), and 70°C (◆). (b) GABA titer at 12 hr of bioconversion period of cytosolic system at 30°C and surface-displayed system at various temperature ranges at 30, 40, 50, 60, and 70°C.

Considering the hyperthermophilic nature of *P. horikoshii*, we investigated the effect of high temperatures. We cultured the recombinant strain with surface-displayed *P. horikoshii* GadB at various temperatures (30, 40, 50, 60, and 70°C). The GABA concentration-time profiles showed a rapid increase during the first 12 hr of the biotransformation period, and then a gradual increase except at 70°C (Fig. 4a). Of the above-listed temperatures, 60°C produced the highest GABA concentration of 5.35 g/l in 24 hr, which is 87.7% of the maximum yield. Also, it produced a high concentration of GABA (4.62 g/l) in 12 hr. Further increasing the temperature to 70ºC decreased the final GABA concentration to 1.36 g/l.

The notable point of the data is that the initial GABA productivity varied significantly as the temperature changed while every tested temperature yielded similar final amounts of GABA. This indicates that, as expected, the reaction rate of the MSG to GABA conversion or the GABA productivity can be improved by elevating the temperature. By increasing the temperature from 30 to 60°C, the GABA productivity during the first 12 hr increased by 66% and the GABA yield increased from 52.8 to 87.7% (Fig. 4b). These results demonstrate the benefit of using hyperthermophilic enzymes in bioprocesses.

One of the biggest challenges for the industrialization of biorefinery production is cost-effectiveness. Besides the final GABA concentration, GABA productivity is one of the key factors because a higher GABA productivity leads to shorter process times and lower production costs. In this study, we increased GABA productivity by employing two strategies. The GABA productivity rate of displayed GadB was higher than that of cytosolic GadB with respect to the bioconversion period. We further increased GABA productivity by 66% via elevating temperature. In total, we increased GABA productivity by a factor of 12.7. Therefore, we believe that our strategy improved the GABA process, and this study can contribute to the development of new high-yield GABA-producing strains at elevated temperatures.

## Funding

This work was financially supported by the National Research Foundation of Korea (NRF) grant funded by the Korea Government (MSIT) (2020R1A2C1008840).

## Conflict of Interest

Authors of this article declare no conflict of interest.

## Data Availability

Experimental data are provided in the manuscript. Authors agree to provide any other data if requested.
